# Reproducibility of a novel, vacuum‐assisted immobilization for breast stereotactic radiotherapy

**DOI:** 10.1002/acm2.13127

**Published:** 2021-03-03

**Authors:** James W. Snider, Elizabeth M. Nichols, Yildirim D. Mutaf, Shifeng Chen, Jason Molitoris, Tejan Diwanji, Stewart J. Becker, Steven J. Feigenberg

**Affiliations:** ^1^ Department of Radiation Oncology University of Alabama at Birmingham Alabama Birmingham AL USA; ^2^ Department of Radiation Oncology University of Maryland School of Medicine Baltimore MD USA; ^3^ Department of Radiation Oncology Kaiser Permanente Dublin CA USA; ^4^ Department of Radiation Oncology University of Miami Coral Gables FL USA; ^5^ Department of Radiation Oncology University of Pennsylvania Philadelphia PA USA

**Keywords:** breast cancer, stereotactic radiotherapy

## Abstract

A novel, breast‐specific stereotactic radiotherapy device has been developed for delivery of highly conformal, accelerated partial breast irradiation. This device employs a unique, vacuum‐assisted, breast cup immobilization system that applies a gentle, negative pressure to the target breast with the patient in the prone position. A device‐specific patient loader is utilized for simulation scanning and device docking. Prior to clinical activation, a prospective protocol enrolled 25 patients who had been or were to be treated with breast conservation surgery and adjuvant radiotherapy for localized breast cancer. The patients underwent breast cup placement and two separate CT simulation scans. Surgical clips within the breast were mapped and positions measured against the device’s integrated stereotactic fiducial/coordinate system to confirm reproducible and durable immobilization during the simulation, treatment planning, and delivery process for the device. Of the enrolled 25 patients, 16 were deemed eligible for analysis. Seventy‐three clips (median, 4; mean, 4.6; range, 1–8 per patient) were mapped in these selected patients on both the first and second CT scans. X, Y, and Z coordinates were determined for the center point of each clip. Length of vector change in position was determined for each clip between the two scans. The mean displacement of implanted clips was 1.90 mm (median, 1.47 mm; range, 0.44–6.52 mm) (95% CI, 1.6–2.20 mm). Additional analyses stratified clips by position within the breast and depth into the immobilization cup. Overall, this effort validated the clinically utilized 3‐mm planning target volume margin for accurate, reliable, and precise employment of the device.

## 1 INTRODUCTION

Although breast cancer remains the most commonly diagnosed malignancy among women in the United States, substantial advances in therapy have been achieved over the last several decades.^1^ In particular, widespread and effective breast cancer screening programs have caused a significant stage migration toward earlier diagnosis, which has translated to improved prognoses.^2^ With such impressive outcomes in early‐stage disease, innovations have focused on reducing the morbidity and improving convenience and healthcare costs associated with surgical, systemic, and radiation therapies for breast cancer.

Breast conservation therapy has emerged as the preferred approach to mastectomy, with comparable disease control and improved quality of life.^3^ This approach has traditionally employed lumpectomy (partial mastectomy) or removal of the breast tumor with a small rim of normal surrounding breast tissue, followed by adjuvant radiotherapy. Breast radiotherapy has most often been delivered to encompass the whole ipsilateral breast tissue (i.e., whole‐breast radiotherapy [WBRT]) while avoiding deep underlying organs at risk. Standard fractionated radiotherapy involved 5.5–6 weeks of treatment. In an effort to reduce side effects and improve quality of life related to radiotherapy, accelerated partial breast irradiation (APBI) has been advocated in a select group of patients.^4^, ^5^, ^6^ This has proven as effective as WBRT while substantially reducing the volume of tissue irradiated and the duration of therapy to 1 week or less depending on the technique of therapy.^7^, ^8^, ^9^, ^10^, ^11^, ^12^

External‐beam radiotherapy (EBRT) has become the technique preferred by both patients and clinicians for delivery of APBI.^13^ This is probably the result of its familiarity to physicians, ease of use, completely noninvasive nature, and wide availability. This has most commonly been used in clinical trials with three‐dimensional conformal radiotherapy (3DCRT) techniques. Early reports from these trials indicate that 3DCRT APBI, although similar in oncologic efficacy to WBRT, has produced unexpectedly high rates of fair/poor cosmetic outcomes.^14^, ^15^, ^16^ Dosimetric analyses from several single‐institution experiences implicated the volume of normal breast tissue exposed to each of several dose prescription levels as predictive of these fair/poor cosmeses.^14^, ^15^

At our institution, several strategies have been explored to reduce normal breast tissue exposure during APBI. We have previously reported our institutional results utilizing preoperative 3DCRT APBI to decrease target volume, because the in vivo tumor is uniformly and powers of magnitude smaller than the eventual lumpectomy cavity with margin.^17^, ^18^ This work led to a prospective, phase II clinical trial of preoperative 3DCRT APBI with excellent disease control rates so far and promising cosmetic outcomes.^19^ In parallel, a novel breast‐specific stereotactic radiotherapy (BSRT) device (GammaPod; Xcision Medical Systems, LLC, Columbia, MD) was developed at our academic medical institution. This device employs non‐overlapping, non‐coplanar ^60^Co beams that rotate around the breast as the patient, in the prone position, is translated over the apertures employing a dynamic dose‐painting technique.^20^, ^21^, ^22^, ^23^, ^24^, ^25^ We have recently reported in silico results demonstrating improvements in dose fall‐off and conformity in comparison with traditional APBI techniques (e.g., 3DCRT, intensity‐modulated radiation therapy, etc).^26^, ^27^ The purpose of this study was to establish the setup accuracy for this stereotactic system that allows for a substantial reduction in planning target volume (PTV) for APBI. The BSRT delivery system is coupled with a device‐specific, vacuum‐assisted breast immobilization cup by which a slight, comfortable negative pressure (150 mm Hg) is applied to the breast and through which a stereotactic registration fiducial system is established. This cup can be registered to both the simulation and treatment tables. Prior to activation of the BSRT device on clinical trial, we completed this reproducibility study for the immobilization system and report the results here.

## 2 MATERIALS AND METHODS

Twenty‐five patients were enrolled on a prospective Institutional Review Board–approved protocol (GCC 1047) at the University of Maryland School of Medicine in the Greenebaum Comprehensive Cancer Center. Eligible patients were those who had previously undergone, were planned to undergo, or were undergoing WBRT by EBRT following partial mastectomy and who could tolerate the prone position.

**FIG. 1:**
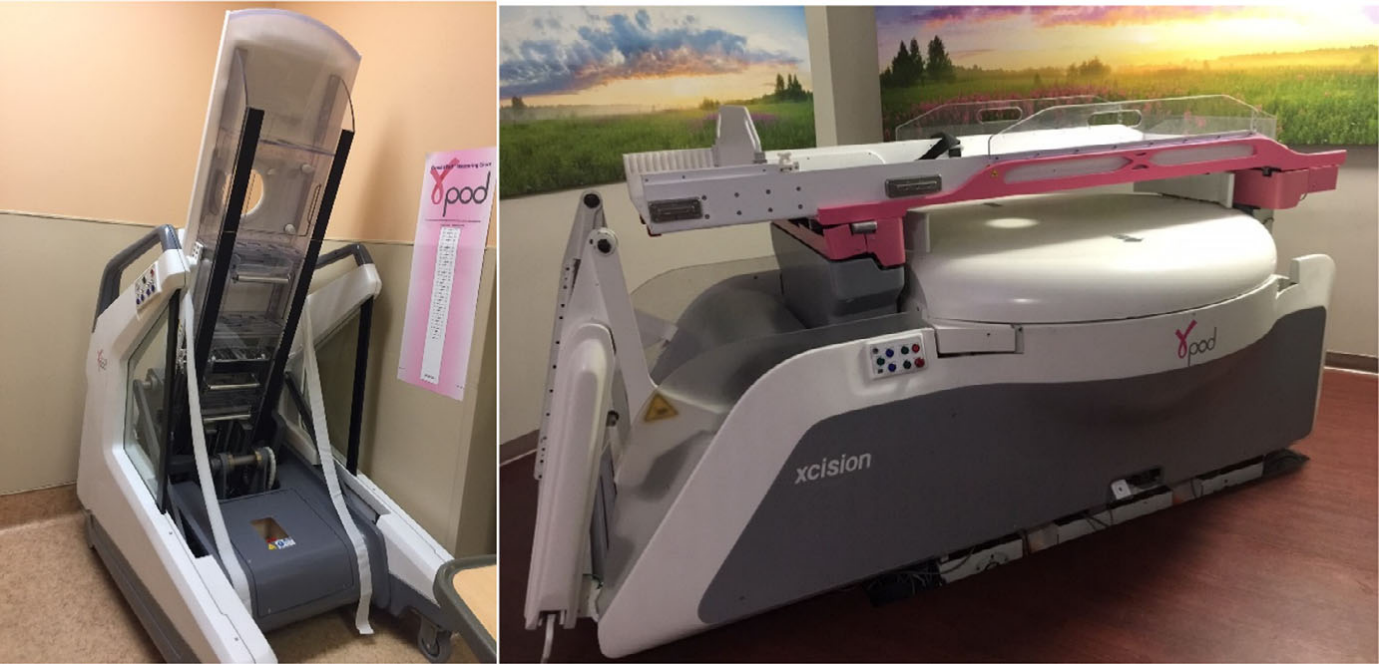
Breast stereotactic radiotherapy device

One physician completed breast‐cup fitting on all participating patients. The three outer cup sizes (small, medium, and large) vary in the width of the base (Fig. 1). Each outer cup, in turn, has 9 or 10 sets of inner cups with varying height (apex‐to‐base) sizes. The outer cup also has an incorporated fiducial system that acts to establish the stereotactic coordinate system. The edge of the inner cup is inserted into a groove along the rim of the silicon flange, which then also locks into the outer cup, forming the combined breast cup immobilization device. The area between the inner and outer cups is subjected to negative pressure with a vacuum device. Through the perforations on the inner cup, the breast is subjected to the gentle pulling of negative pressure and fills the inner cup volume. This immobilizes the breast tissue within the cup. This system has been described previously in greater detail.^21^

Despite the application of negative pressure, the anatomy of some patients did not allow the entire breast to fill the inner cup because the cup’s geometry reflects a regular and round surface. This was exacerbated in patients who had prior radiation and some loss of breast elasticity. Where large (>1 cm) gaps were visible between the breast and inner cup, a silicone filler was inserted into the inner cup to close the gap. The silicone fillers are akin to commercially available silicone bra inserts and are intended to comfortably fill negative space within the inner cup with appropriate rigidity; size/fitting for these was individualized. This was adhered to the inner cup with adhesive prior to repeat placement of the cup.

Once the breast cup was fitted and the vacuum applied, each patient was placed on the device‐specific image loader in the upright position. The patient was then loaded onto the CT simulation table from the standing to prone position. The patient loader lays the device‐specific table on top of the native CT table rather than scanner‐specific docking. The breast cup was positioned through a hole in the loader to which the breast cup was registered and locked. The patient underwent CT (CT1) imaging in this position, with 1‐mm slice thickness. Each patient was then removed from the CT table by the loader and asked to remain in the breast cup for 30 min to simulate treatment planning time and transport time to the device for delivery. Patients were, then, again loaded onto the CT table and rescanned (CT2). As most applications of this device are planned to be single fraction implementations, this workflow was felt to adequately simulate actual treatment preparation, simulation, and delivery. In potential multi‐fraction applications of the device, the cup would be re‐fitted and the patient resimulated for each delivery; therefore, interfractional variability in breast cup placement is of less concern.

Both CT simulations were transferred to the institutional treatment planning software. A physician contoured each surgical clip as a separate region of interest on both CT1 and CT2, and each clip was numbered to correlate clips between scans. These clips were used as surrogates for assessing position of the postoperative bed in this study. Note that surgeons were not instructed as to clip placement as patients for this protocol were selected after all oncologic interventions. Standardly in clinical practice at our institution, breast surgeons place a limited number of clips to aid with operative bed identification for radiotherapy and clinical follow‐up. Both DICOM datasets were then transferred to the BSRT dedicated planning software for analysis. Images were registered based on the breast cup’s radio‐opaque helical fiducial wire, which has a well‐documented geometry. The fiducial wire facilitates construction of the global stereotactic coordinate system for BSRT treatments. Through a rigid registration in the treatment planning system, a 3D transformation between the imaging coordinate system and the stereotactic coordinate system is created. Because of the unique shape of the wire, this registration also allows the treated breast site to be automatically recognized from the images. When the breast cup is engaged onto the BSRT treatment table, the stereotactic coordinate frame on the cup then becomes the coordinate system used by the treatment unit, therefore linking the radiation isocenter coordinate to patient anatomy.

The center point of each delineated clip was localized manually within the BSRT treatment planning system. With this methodology and considering the 1‐mm thickness of CT slices, an approximate and inherent 1‐mm error with random distribution is assumed. *X*, *Y*, and *Z* coordinates were recorded for both CT1 and CT2. The displacement of each clip from the first CT exam to the second was calculated as a vector following registration of the two scans based on the cup’s integrated stereotactic fiducial. Clips were categorized as being either “within the cup” (below the surface of the table) or “above the cup” (closer to the chest wall) to ensure that breast tissue outside the cup but within the target limitations of the BSRT geometry was also reasonably immobilized with this technique. Clips in the axilla, rather than the lumpectomy cavity, were eliminated from the analysis. In addition, patients with a tumor bed/clips more than 1 cm outside the breast cup (above the treatment table) were omitted from final analysis, because these targets would be outside the geometric limitations of the BSRT device system.

## 3 RESULTS

Of the initial 25 patient volunteers, nine were deemed ineligible for this analysis based on aborted procedures prior to CT2, clips outside of the physical treatment limitations of the device’s geometry, clips deemed mobile within the postoperative seroma, or lack of implanted tumor bed clips (Table 1). The remaining 16 patients were analyzed (Table 2). Ineligible patients were utilized in separate theoretical planning studies and reports for dosimetric evaluation of the BSRT device.^26^, ^27^ Seventy‐three clips (median, 4; mean, 4.6; range, 1–8 per patient) implanted in the selected 16 patients were utilized for assessment of immobilization reproducibility.

**TABLE 1:** Patient demographics

Patient no.	Age (y)	RT status	Breast laterality	Breast quadrant	Bra size	Outer cup selection	Inner cup selection	Imaging performed	Clips appropriate for study	Silicone filler	Significant pressure loss
1	53	After	Left	UIQ	36DD	Large	1	Yes	Yes	No	No
2	46	After	Left	C	42D	Large	1	Yes	Yes	No	No
3	68	After	Right	UOQ	40D	Large	1	Yes	Yes	No	Yes
4	71	After	Left	UIQ	34B	Medium	1	Yes	Yes	No	No
5	77	After	Right	UOQ	38B	Medium	2	No^1^	Yes	N/A	N/A
6	63	After	Left	UOQ	42C	Large	5	No^3^	Yes	N/A	N/A
7	41	After	Left	C	36C	Large	1	Yes	Yes	Yes	No
8	80	After	Right	UOQ	34B	Medium	3	Yes	Yes	Yes	No
9	73	After	Left	UOQ	N/A	N/A	N/A	No^2^	Yes	N/A	N/A
10	44	After	Right	UOQ	38D	Large	1	Yes	Yes	Yes	No
11	46	After	Right	LOQ	34B	Medium	1	Yes	No^4^	Yes	No
12	41	After	Left	UIQ	34B	Medium	1	No^3^	Yes	N/A	N/A
13	70	After	Right	UOQ	36B	Medium	1	Yes	Yes	Yes	No
14	71	During	Left	LOQ	36B	Medium	2	Yes	Yes	Yes	Yes
15	62	After	Left	UIQ	40C	Large	1	Yes	No^4^	Yes	Yes
16	56	After	Right	UOQ	38B	Medium	1	Yes	Yes	Yes	No
17	48	After	Right	UIQ	36B	Medium	4	Yes	Yes	No	No
18	62	After	Right	LIQ	36A	Medium	1	Yes	Yes	Yes	No
19	53	After	Left	LOQ	36B	Medium	1	Yes	Yes	Yes	Yes
20	76	After	Left	C	44C	Large	1	Yes	No^4^	Yes	Yes
21	64	After	Right	LIQ	38B	Medium	1	No^3^	Yes	N/A	N/A
22	59	Before	Right	C	38C	Medium	2	Yes	Yes	No	Yes
23	47	Before	Left	UIQ	40C	Large	1	Yes	Yes	Yes	No
24	66	Before	Right	UIQ	40B	Large	1	Yes	Yes	Yes	No
25	‐‐	‐‐	Left		‐‐	Medium	7	Yes	No^5^	No	No

All bra sizes are noted as standard United States sizes and as reported by the patient.

UIQ,, upper inner quadrant; UOQ, upper outer quadrant; LIQ, lower inner quadrant; LOQ, lower outer quadrant; C, central.

^1^ Pressure seal was not obtained as a result of patient body habitus.

^2^ No appropriate breast cup size was found.

^3^ Procedure was aborted because of discomfort.

^4^ No appropriate clips for vector measurement.

^5^ Clips mobile within a lumpectomy seroma.

**TABLE 2:** Mean, median, and range of vectors of displacement (in mm) of clips for each assessable patient (*n* = 16)

Patient no.	Mean clip displacement	Median clip displacement	Range clip displacement	Lost pressure
1	2.7	3.0	1.69–3.69	
2	2.1	2.1	1.4–2.7	
3	2.2	1.6	1.3–4.1	X
4	1.5	1.7	0.9–1.8	
7	0.8	0.8	0.5–1.2	
8	0.9	0.9	0.5‐1.5	
10	1.6	1.6	1.0–2.3	
13	1.3	1.3	0.7–2.3	
14	2.6	2.5	2.3–3.0	X
16	3.0	2.4	1.5–7.2	
17	1.4	1.4	1.4–1.4	
18	0.9	0.9	0.7–1.1	
19	4.1	4.1	1.3–6.5	X
22	0.7	0.5	0.4–1.3	X
23	2.3	1.0	0.8–5.6	
24	1.9	1.8	0.7–3.4	

Of the 16 patients, 12 completed the entire protocol with no evidence of breast cup pressure loss. Early in this protocol, several modifications were made as experience with breast cup placement, cup filling, and flange application increased among the treating physicians. These alterations have subsequently greatly improved the reliability of the pressure seal with the device. However, four patients were included in this analysis who had only momentary pressure decrease or loss so that the effect of momentary seal loss on reproducibility could be characterized. The current clinical practice, however, is to rescan the patient if a momentary pressure loss occurs between scan and treatment, because it is impossible to be certain that the stereotactic coordinates remain unchanged.

**FIG. 2:**
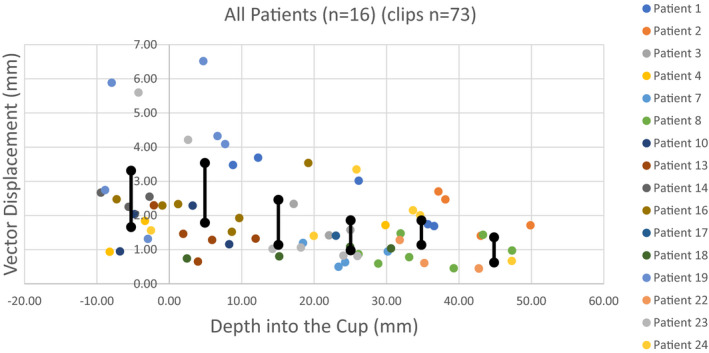
Entire cohort clip displacement distance from CT1 to CT2 for individual clips (*n* = 16 patients, 73 clips), with 95% CIs (black lines)

For the entire cohort (*n* = 16), the mean displacement of each clip (*n* = 73) was 1.90 mm (median, 1.47 mm; range, 0.44–6.52 mm) (Fig. 2). The 95% confidence interval (CI) extended from 1.61 to 2.20 mm (SD, 1.28). Clips were included in the analysis if they fell within the breast cup or within 1 cm outside the cup, because these remain within the physical limitations of the device and, therefore, would be clinically treatable locations. Of the 73 clips, 58 were within the cup and, therefore, below the surface of the treatment table (Table 3). For clips within the cup the mean displacement was 1.75 mm (median, 1.41 mm; range, 0.44–6.5 mm; 95% CI 1.44–2.06; SD, 1.21). For clips above the cup (*n* = 15) but within 1 cm from the table surface, the mean displacement was 2.49 mm (median, 2.29 mm; range, 0.94–5.88 mm; 95% CI 1.79–3.2; SD, 1.39).

**TABLE 3:** Mean, median, and range of clip (n = 73) displacements by depth (in mm) in breast cup

Depth in breast cup	Mean	Median	Range
−1–0 (outside cup)	2.5	2.3	0.9–5.9
0–1	2.6	2.3	0.6–6.5
1–2	1.8	1.3	0.8–3.7
2–3	1.4	1.2	0.5–3.3
3–4	1.5	1.5	0.5–2.7
4–5	1.0	1.0	0.4–1.7

**FIG. 3:**
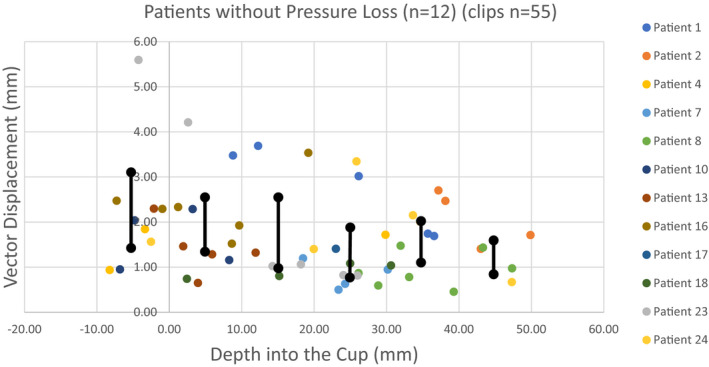
Individual clip displacement distance from CT1 to CT2 for patients without cup pressure loss (*n* = 12 patients, 55 clips), with 95% CIs (black lines)

For the 12 patients without noted pressure loss during the procedure, the mean displacement of each clip (*n* = 55) was 1.70 mm (median, 1.43 mm; range, 0.45–5.60 mm; 95% CI 1.43–1.97; SD, 1.03) (Fig. 3). Of these, 46 clips were within the cup, and these clips changed in position, on average, by only 1.60 mm (median, 1.40 mm; range, 0.45–4.2 mm; 95% CI 1.33–1.87; SD, 0.93) (Table 4). For the nine clips outside the cup in patients without pressure loss, mean positional change was 2.22 mm (median, 2.04 mm; range, 0.94–5.60, mm; 95% CI 0.85; SD, 1.31).

**TABLE 4:** Mean, median, and range of clip (n = 55) displacement (in mm) by depth in breast cup for patients without pressure loss

Depth in breast cup	Mean	Median	Range
–1–0 (outside cup)	2.2	2.0	0.9–5.6
0–1	1.9	1.5	0.6–4.2
1–2	1.8	1.3	0.8–3.7
2–3	1.3	0.9	0.5–3.3
3–4	1.6	1.7	0.5–2.7
4–5	1.2	1.4	0.7–1.7

**FIG. 4:**
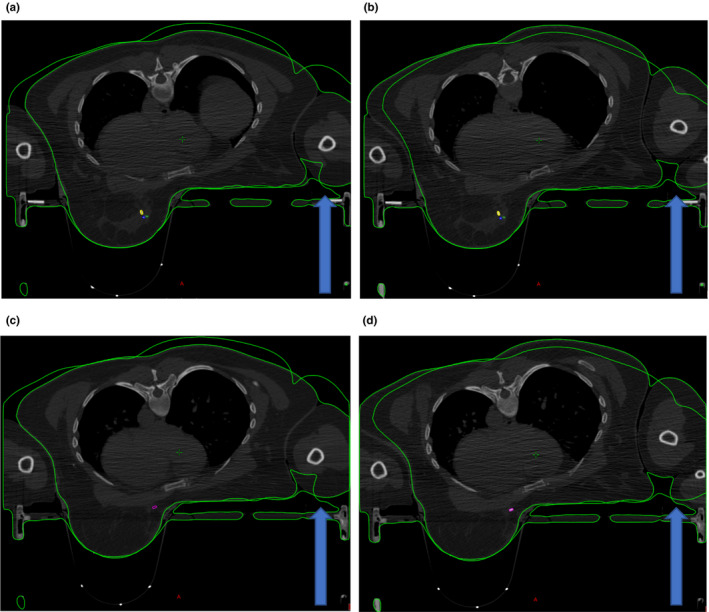
Patient with substantial positioning difference between CT1 and CT2 examinations. Note the folded right arm (blue arrow) in CT2 (b and d) vs. initial CT1 position (a and c) on two representative axial slices (a vs. b, c vs. d), which has substantially changed the external contour (green, all images) and rotated the patient. Also note that the clips immediately surrounding the lumpectomy cavity remain relatively unchanged (a and b)

It should be noted that only one patient without pressure loss had a clip change in position by more than 4 mm. This patient had two clips (displaced 5.6 and 4.2 mm) that were more distant from the lumpectomy cavity and tethered to the chest wall. In comparing the two CTs, the patient’s arm position was quite different, causing a substantial rotation (Fig. 4). Despite this, the patient’s four clips more proximate to the lumpectomy cavity had vectors of 1.06 mm or less (0.81, 0.82, 1.02, and 1.06 mm). All other patients (*n* = 3) with clips displaced more than 3 mm also had other clips that were less than 3 mm from their original positions.

## 4 DISCUSSION

This BSRT device represents a novel, breast stereotactic radiotherapy system with two key advantages over other external‐beam delivery platforms: a unique dynamic dose‐painting delivery technique and a device‐specific breast stereotactic immobilization cup. Based on results from the current study, the immobilization cup offers excellent reproducibility both within and above the cup’s brim (at level of treatment table top) up to 1 cm, which coincides with the geometric limit of the device’s delivery. Our analysis of internal marker localization accuracy indicates that 86% of markers could be localized with an uncertainty of less than 3 mm. In patients without breast cup pressure loss or substantially different arm position at CT2, 93% of clips were displaced less than 3 mm. In this group, all (100%) clips were displaced less than 4 mm. Due to limitations of the treatment planning software in localization of clip center points and the inherent slice thickness of CT, an inherent error of at least 1 mm could be expected on each clip. As such, outliers of between 3 and 4 mm of difference, as were encountered in this series, are expected and should not influence setup uncertainty corrections/recommendations. That with proper setup and lack of pressure loss, all clips were within 4‐mm difference is reassuring.

Although PTV margin should take into account other applicable uncertainties (mechanical and dose delivery uncertainties, etc.), localization uncertainty is deemed a major component driving PTV margin. As such, we have concluded that it is reasonable to utilize a PTV margin of 3 mm in treatment planning with this device. This is substantially less than the 10 mm recommended on previous APBI trials.^13^, ^16^

It should be noted that for cases in which the target is close to or extends slightly above the treatment table and, therefore, outside of the immobilization cup, care must be taken to prevent differences in patient positioning between simulation and treatment specifically as it pertains to arm positioning. Such changes can lead to significant alterations in target positioning, as seen in Fig. 4. For targets that extend outside the cup, additional upper‐body or thoracic immobilization techniques, which were not included in this study, might be considered. In addition, a room or unit‐mounted laser localization or optical guidance system could also be utilized to improve the reproducibility of upper‐body positioning.

A relatively high incidence (7/25% or 28%) of pressure loss was observed during the study. Some factors associated with this observation may be the strength of the medical adhesive used on the silicone flange as well as the contours of patients’ bodies. Early in this study, we noted that the strength of the original adhesive used on the silicone flange was insufficient and, therefore, not effective in maintaining an adequate seal around the breast. This was partially remedied with additional spraying of medical‐grade adhesive on the flange to strengthen the seal. This step has substantially lessened the frequency of pressure loss, but additional improvements have been made to optimize prolonged seal.

Most of the pressure loss incidents occurred during the loading or unloading stages of the imaging when the patient mounted the treatment couch in the vertical position and as transitioned to prone position on the scanner. Additional instructions and support have subsequently been provided to the patient to minimize pressure loss during this critical patient movement. It is anticipated that pressure integrity should not be compromised during image acquisition or treatment delivery because of the prone positioning and natural pressure with gravity of the body against the flange, augmenting the seal. However, we conservatively repeat pretreatment imaging and restart planning in cases of pressure loss. In addition, following this effort, we have recommended that patient arm position be verified as relatively similar between imaging and treatment.

In this prospective study the immobilization technique and breast cup system were well tolerated by patients, with generally minimal discomfort or side effects. Small, asymptomatic superficial skin blisters were encountered in one case that self‐resolved. Otherwise, the majority of patients underwent CT1, waited for 30 min, and underwent CT2 without significant issue or pain.

In summary, limiting the exposure of normal tissue to radiation dose is appropriately prioritized in patients with early‐stage breast cancer, where high rates of cure predominate. While keeping local control rates relatively similar to whole‐breast irradiation, APBI approaches can offer measurable reductions in dose to normal tissues, including the lung, heart, chest wall, breast skin, and uninvolved breast tissue. As previously noted, several efforts have detailed unexpectedly poor cosmetic outcomes with APBI delivered with traditional EBRT techniques.^14^, ^15^, ^16^ These outcomes have been clearly linked to the amount of normal breast and skin tissue exposed to radiotherapy. In silico work at our institution has demonstrated improved dose conformality and substantial reductions in breast and other organ‐at‐risk exposure with the BSRT device.^26^, ^27^ The current work has verified the appropriateness of margins utilized for setup uncertainty. The device has been activated clinically at two institutions with four additional sites projected within the next 2 years.^22^, ^23^, ^24^, ^25^, ^28^

## 5 CONCLUSION

The device‐specific negative‐pressure breast cup evaluated here offers excellent immobilization and reproducibility, with an average setup uncertainty of ≤3 mm. This serves as the recommended PTV margin for utilization of the device on currently activated and planned clinical trials as well as in general clinical practice. Further work is underway to improve manufacturing and application of the immobilization cup to prevent pressure losses and further reduce uncertainty.

## Conflicts of interest

Snider reports conflicts related to the Maryland Industrial Partnerships grant which has funded device development and research for the GammaPod device. Feigenberg and Nichols report the same conflict. Nichols and Becker report speaking for and representing Xcision Medical Systems who developed and market the GammaPod device. Nichols is the primary investigator for clinical trials investigating the application of the GammaPod device.

Snider reports unrelated conflicts including honorarium, consulting, and travel expenses from Varian Medical Systems, Siemens Healthineers, and the Society for Thermal Medicine. Snider reports an unrelated patent regarding a proton radiotherapy planning methodology.

All above sources of bias have been effectively mitigated in this investigation through systematic methodology for data collection and analysis.
